# Exploring cost reduction strategies for serum free media development

**DOI:** 10.1038/s41538-024-00352-0

**Published:** 2024-12-21

**Authors:** Jun Ping Quek, Azra Anwar Gaffoor, Yu Xuan Tan, Tessa Rui Min Tan, Yu Feng Chua, Dawn Sow Zong Leong, Alif Sufiyan Ali, Say Kong Ng

**Affiliations:** Bioprocessing Technology Institute (BTI), Agency for Science, Technology and Research (A*STAR), 20 Biopolis Way, #06-01 Centros, Singapore, 138668 Republic of Singapore

**Keywords:** Biochemistry, Biotechnology, Cell biology, Molecular biology

## Abstract

Cultivated meat production offers solutions in addressing global food security and sustainability challenges. However, serum-free media (SFM) used in cultivating the cells are expensive, contributing to at least 50% of variable operating costs. This review explores technologies for cost-effective SFM, focusing on reducing cost from using growth factors and recombinant proteins, using affordable raw materials for basal media, and implementing cost-saving measures like media recycling and reducing waste build-up.

## Introduction

With a global population projected to reach 9.6 billion by 2050, the United Nations forecasts a need to increase food production by 70% to meet this demand^1^. Globally, it is also projected that poultry, pork, beef, and lamb consumption will increase by 10–15% by 2032 compared to 2023 levels^2^. Cultivated meat (CM), which requires much less land use compared to traditional farming, thus serves as a promising alternative protein source. CM, produced by growing animal cells in bioreactors, is a growing industry with a market size valued at $221.47 million in 2022 and estimated to reach $592.69 million by 2030^3^. The demand for CM is likely to increase with growing interests in sustainable protein sources for environmental and animal welfare reasons. However, one major obstacle that the industry is currently facing is the high cost of production.

**Fig. 1:**
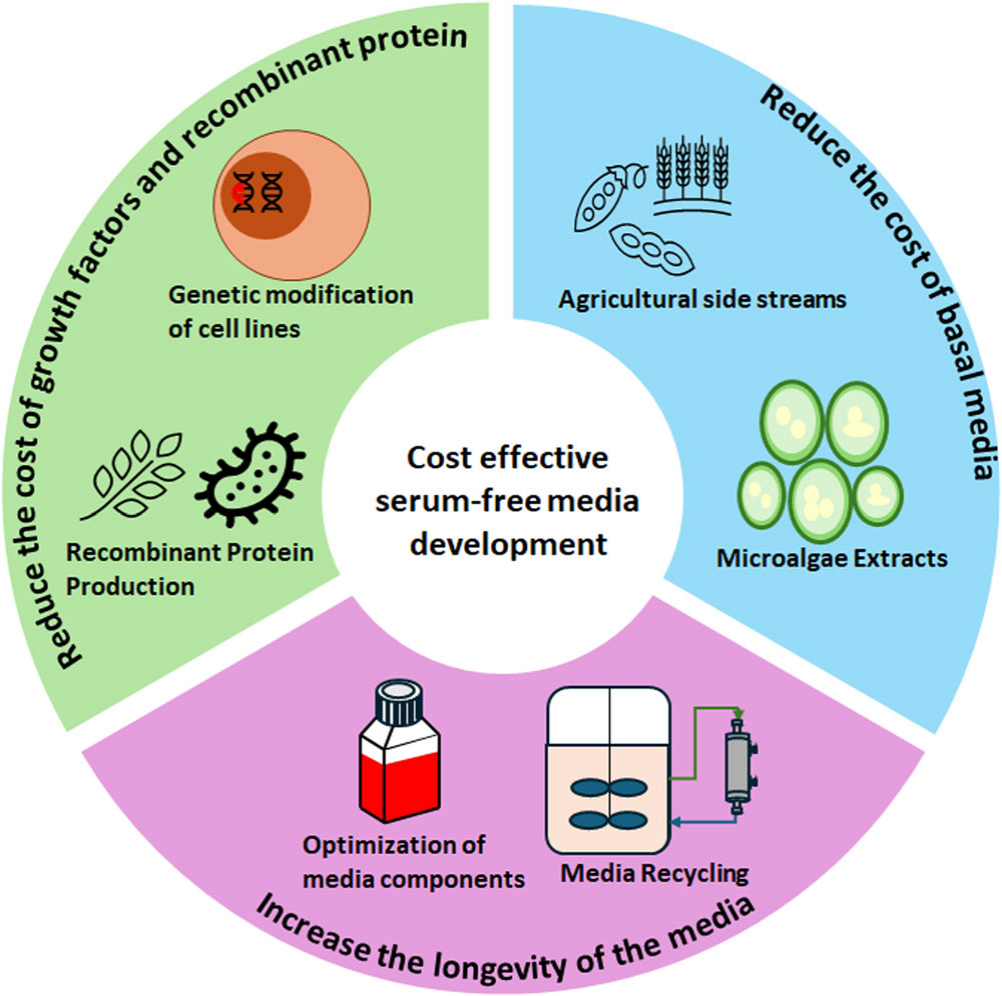
Current technologies to reduce the cost of serum-free media (SFM) in cultivated meat production. Strategies include lowering the cost contributions of growth factors, recombinant proteins, and basal media. Additionally, optimizing media components and recycling SFM can enhance media utilization and reduce costs associated with media changes.

The cultivated meat industry is increasingly adopting serum-free media (SFM) to address the cost, ethical, safety, and regulatory concerns associated with using fetal bovine serum (FBS)^4–6^. Currently, SFM makes up at least 50% of variable operating costs in cultivated meat manufacturing^7^, mainly due to the use of growth factors (GFs) and recombinant proteins (RPs)^8–11^. For example, in the Essential 8 medium, nearly 98% of the media cost can be attributed to FGF-2 and TGF-β^9^. Similarly, the cost of the SFM beefy-9 is largely driven by key components like albumin, FGF-2, and insulin, collectively representing around 60% of the total media cost^10^. Advancements in SFM technology will be major drivers for cultivated meat to reach price parity with conventional meat^9,12,13^. This review will examine current technologies for developing cost-effective SFM tailored to cultivated meat production, such as reducing cost of growth factors and recombinant proteins, using affordable raw materials for basal media, and optimizing media composition and usage **(**Fig. 1**)**.

### Methods for serum-free media development

This section will explore common methodologies for developing SFM, including substituting FBS with known components, media component screening, omics analysis and systems biology approaches.

Firstly, SFM can be developed by substituting FBS with known growth-promoting components, such as growth factors, insulin, transferrin, and selenium. For instance, Mosa Meat developed a SFM by replacing FBS with major and known components of FBS into Ham’s F10 basal medium^8^. Separately, Stout and his team improved upon the reported SFM B8^14^ by developing Beefy-9, which adds recombinant albumin and reduces growth-factor concentrations^10^. The Beefy-9 media could support long-term cell culture while retaining myogenic potential^10^. Elsewhere, Skrivergaard’s team used multi-component Design of Experiment (DOE) to screen for components and growth factors that enhanced proliferation of bovine satellite cells^11^. Through multiple DOE rounds and response surface methodology for optimization, they developed a SFM containing fetuin, bovine serum albumin, FGF2, and an insulin-transferrin-selenium supplement^11^.

In addition to testing known growth-promoting components, omics approaches can be effectively utilized to develop SFM. For instance, Lin and colleagues used intracellular metabolomics analyses to select candidate growth-promoting metabolites that could account for the differences in chicken fibroblasts’ growth profiles seen in two different basal media^15^. Using DOE, they achieved 40.72% higher cell growth by optimizing the concentrations of 28 candidate metabolites^15^. In another study, Messmer et al. employed a transcriptomic approach to identify surface receptors upregulated during myogenic differentiation induced by serum starvation^16^. By testing the corresponding ligands, they formulated a serum-free myogenic differentiation medium^16^. Next, Gomez Romero and Boyle’s review^17^ highlights the use of systems biology and metabolomics tools to understand key metabolic pathways and genes^17^. These insights can be applied to media design and bioprocess optimization, potentially lowering cell-based meat production costs^17^. By understanding the metabolic and nutrients requirements of the human induced pluripotent stem cells, Lyra-Leite and colleagues managed to optimize and reduce the number of media components in B8^14^ media down to 39 components^18^.

While there have been successes in developing SFM for CM cell lines, studies reveal that nutrient requirements vary across different species and cell types^17,19^. This indicates that a one-size-fits-all SFM is unlikely to be effective for culturing multiple cell types. Additionally, variations in cellular metabolism at different stages of differentiation may necessitate optimizing SFM for both the cell growth and differentiation phases, requiring different formulations throughout the cultivated meat production process^20^. Therefore, SFM development pipelines must be robust enough to create tailored media formulations specific to the various cell lines and cell states in the CM industry.

### Serum-free media for cultivated meat

Several cultivated meat companies, including Mosa Meat^8,16,21^, GOOD Meat^22^, Upside Foods^23^, Aleph Farms^24^, Believer Meat^25,26^, Vow^27^, and CellMeat^28^, have successfully developed SFM. Additionally, there are also a myriad of commercially available serum replacements for cultivated meat cell lines produced by various companies such as ClearX9 produced by Clear Meat^29^, Multus Biotechnology’s Proliferum M^30^, NouBio’s NouSerum^31^ and microorganism-based growth medium supplement by Biftek.co^32^. Currently, several serum-free cultivated meat products have received regulatory approval in multiple countries. In January 2023, the Singapore Food Agency approved GOOD Meat’s serum-free cultivated chicken for production in Singapore^22^. In January 2024, Israel’s Ministry of Health approved Aleph Farms’ serum-free cultivated beef^24^, followed shortly by Singapore’s approval of Vow’s serum-free quail product^27^. Most recently, in July 2024, Meatly received regulatory clearance to produce its cultivated pet food in the United Kingdom^33^.

Several SFM formulations are also published and summarized in Table 1. A comparison of these formulations showed that DMEM/F-12 was commonly used as the basal media, as it combined the high nutrient content of DMEM with the diverse components of Ham’s F12. FGF2 was frequently employed as a growth factor, with insulin and transferrin also common media additives.

**Table 1:** Published serum-free media formulation with concentrations for cultivated meat application

Media Types	Growth Media				Differentiation Media	
Cell Types	Primary Bovine Satellite Cells^8^	Primary Bovine Satellite Cells^10^	Primary Bovine Satellite Cells^11^	Primary chicken fibroblasts cells^25^	Primary Bovine Satellite Cells^16^	Bovine, ovine, porcine, and murine adipogenic precursor cells^21^
Component						
**Basal Medium**						
DMEM/F-12	1 Liter	1 Liter	1 Liter	1 Liter	1 Liter	1 Liter
**Proteins/ Peptides**						
Fibroblast growth factor (FGF-2)	10 ng/mL	40 ng/mL	2 ng/mL	10 ng/mL	—	2 ng/mL
Insulin	—	20 μg/mL	—	3 μg/mL	10.4 μg/mL	10 μg/mL
Transferrin	—	20 μg/mL	—	—	10.8 μg/mL	—
rAlbumin	—	800 μg /mL	—	—	—	—
Bovine serum albumin (BSA)	—	—	75 μg/mL	—	—	—
Human serum albumin (HSA)	5 mg/mL	—	—	—	0.5 mg/mL	—
VEGF	10 ng/mL	—	—	—	—	—
IGF-1	100 ng/mL	—	—	—	—	—
HGF	5 ng/mL	—	—	—	—	—
PDGF-BB	10 ng/mL	—	—	—	—	—
Neuregulin (NRG1)	—	0.1 ng/mL	—	—	—	—
Transforming growth factor (TGFβ3)	—	0.1 ng/mL	—	—	—	—
EGF1	—	—	—	—	10 ng/mL	2 ng/mL
Human IL-6	20 ng/mL	—	—	—	—	—
Fibronectin	10 μg/mL	—	—	—	—	—
Fetuin	—	—	600 μg/mL	—	—	—
BMP4	—	—	—	—	—	10 ng/mL
**Mixtures**						
Insulin-Transferrin-Selenium-Ethanolamine (ITS-X)	10 μg/mL Insulin 5.5 μg/mL Transferrin 6.7 ng/mL Sodium selenite 2 μg/mL Ethanolamine	—	—	—	—	—
ITS	—	—	10 μg/mL Insulin 5.5 μg/mL Transferrin 6.7 ng/mL Sodium selenite	—	—	—
Canola lipid mixture	—	—	—	10 μg/mL	—	—
Lipid concentrate	—	—	—	—	—	0.1%
MEM Amino acid solution	—	—	—	—	0.5%	—
**Steroid/ Hormone/ Drug**						
Hydrocortisone	36 ng/mL	—	—	2 μg/mL	—	9.06 ng/mL
Progesterone	—	—	—	—	—	5.60 ng/mL
Rosiglitazone	—	—	—	—	—	1.78 μg/mL
**Nutrient/ Buffer/ Salts**						
Glucose	—	—	—	—	—	3.06 mg/mL
GlutaMAX™	0.434 g/L	—	—	0.434 g/L	—	—
α-linolenic acid	1 μg/mL	—	—	—	—	—
Lysophosphatidic acid	—	—	—	—	0.437 μg/mL	—
L-ascorbic acid 2-phosphate	50 μg/mL	200 μg/mL	—	—	11.6 μg/mL	65.7 μg/mL
Putrescine	—	—	—	—	—	5.02 μg/mL
HEPES	—	—	—	—	—	1.17 mg/mL
Sodium Bicarbonate	—	—	—	—	68.9 μg/mL	—
Calcium chloride	—	—	—	—	—	0.147 mg/mL
Sodium Selenite	—	20 ng/mL	—	7 ng/mL	13.8 ng/mL	—

A cost analysis of the published formulations at lab-scale is provided in Table 2 and Supplementary Table 1-6. As expected, GFs and RPs account for the majority of the SFM cost, with basal media contributing the remainder. The analysis is based on pricing from life science vendors, but further cost reductions may be achievable through bulk purchasing or sourcing from B2B vendors specializing in the cultivated meat sector. Recently, Believer Meats demonstrated that a serum-free medium can be produced at a cost as low as USD $0.63 per liter^26^, by replacing albumin and optimising concentrations of media components based on the nutritional requirements of the cell. In the following sections, we will discuss potentials in reducing the cost contributions of growth factors, recombinant proteins, and basal media, as well as other strategies to enable more affordable SFM for cultured meat applications.

**Table 2:** Published serum-free media formulation with lab scale cost analysis (SGD) per liter of SFM. The percentage contributions of basal media, proteins/peptides, and other components (mixture, steroids, hormones, salt, buffer, drug,) to the total cost are also shown. For detailed information on the calculation, refer to supplementary table 1-6

Media Types	Growth Media				Differentiation Media	
Cell Types	Primary Bovine Satellite Cells^8^	Primary Bovine Satellite Cells^10^	Primary Bovine Satellite Cells^11^	Primary chicken fibroblasts cells^25^	Primary Bovine Satellite Cells^16^	Bovine, ovine, porcine, and murine adipogenic precursor cells^21^
Component						
**Basal Medium**						
DMEM/F-12 (SGD$)	214.00	212.00	212.00	214.00	214.00	214.00
**Proteins/ Peptides**						
Fibroblast growth factor (FGF-2) (SGD$)	48.00	258.40	6.00	35.60	—	7.12
Insulin (SGD$)	—	41.04	—	15.66	28.19	52.20
Transferrin (SGD$)	—	57.60	—	—	8.09	—
rAlbumin (SGD$)	—	204.00	—	—	—	—
Bovine serum albumin (BSA) (SGD$)	—	—	1.41	—	—	—
Human serum albumin (HSA) (SGD$)	1040.00	—	—	—	155.50	—
VEGF (SGD$)	270.00	—	—	—	—	—
IGF-1 (SGD$)	295.00	—	—	—	—	—
HGF (SGD$)	620.00	—	—	—	—	—
PDGF-BB (SGD$)	710.00	—	—	—	—	—
Neuregulin (NRG1) (SGD$)	—	1.31	—	—	—	—
Transforming growth factor (TGFβ3) (SGD$)	—	6.55	—	—	—	—
EGF1 (SGD$)	—	—	—	—	13.50	2.70
Human IL-6 (SGD$)	317.00	—	—	—	—	—
Fibronectin (SGD$)	1686.00	—	—	—	—	—
Fetuin (SGD$)	—	—	234.60	—	—	—
BMP4 (SGD$)	—	—	—	—	—	160.90
**Mixtures**						
Insulin-Transferrin-Selenium-Ethanolamine (ITS-X) (SGD$)	195.00	—	—	—	—	—
ITS (SGD$)	—	—	132.00	—	—	—
Canola lipid mixture (SGD$)	—	—	—	2.77	—	—
Lipid concentrate (SGD$)	—	—	—	—	—	1.93
MEM Amino acid solution (SGD$)	—	—	—	—	0.07	—
**Steroid/ Hormone/ Drug**						
Hydrocortisone (SGD$)	0.00	—	—	280.00	—	1.27
Progesterone (SGD$)	—	—	—	—	—	0.00
Rosiglitazone (SGD$)	—	—	—	—	—	40.03
**Nutrient/ Buffer/ Salts**						
Glucose (SGD$)	—	—	—	—	—	2.75
GlutaMAX™ (SGD$)	12.90	—	—	15.47	—	—
α-linolenic acid (SGD$)	269.00	—	—	—	—	—
Lysophosphatidic acid (SGD$)	—	—	—	—	34.40	—
L-ascorbic acid 2-phosphate (SGD$)	0.91	3.64	—	—	0.037	2.08
Putrescine (SGD$)	—	—	—	—	—	4.36
HEPES (SGD$)	—	—	—	—	—	5.93
Sodium Bicarbonate (SGD$)	—	—	—	—	0.15	—
Calcium chloride (SGD$)	—	—	—	—	—	0.05
Sodium Selenite (SGD$)	—	0.00	—	0.00	0.00	—
Total Cost/L (SGD$)	$5677.81	$784.54	$586.01	$563.50	$454.26	$495.31
Percentage of cost from Basal Media (%)	3.7	27.0	36.2	38.0	47.1	43.2
Percentage of cost from GF/RPs (%)	91.3	72.5	63.8	9.1	45.2	45.0
Percentage of cost from other components (%)	5.0	0.5	0.0	52.9	7.7	11.8
Total percentage (%)	100	100	100	100	100	100

## Advances to reduce the cost contribution by growth factors and recombinant protein

Given that the cost of SFM is largely attributed to the use of GFs and RPs (Table 2, Supplementary Table 1-6), much of the research focuses on reducing the use of GFs and RPs through genetically engineering cultivated meat cell lines or producing cheaper GFs and RPs.

### Genetic modification of cell lines to produce the required growth factors

The idea of performing genetic modification of cell lines to remove the requirement for GFs in media started as early as 1992. Pietrzkowski et al. randomly integrated both human IGF1 and IGF1R into BALB/c3T3 mouse fibroblasts, finding that the resulting cells could grow in the absence of FBS^34^. Subsequently, another group randomly integrated human IGF1 and transferrin into CHO-K1 cells^35^. These modified cells could then grow in the absence of insulin and transferrin.

In recent years, genetic modifications have also been attempted on cultivated meat cell lines. Stout and colleagues engineered immortalized bovine satellite cells (iBSCs) to express bovine FGF2 and human RAS^G12V^ genes under the control of the Dox-inducible Tet-on promoter^36^. This enabled a doubling time of 60 h, comparable to that of unmodified iBSCs supplemented with recombinant FGF that had a doubling time of 55 hours^36^. The engineered cells were able to proliferate in FGF2-free medium for multiple passages, although with reduced myotube formation during differentiation. Based on this work, Tufts University filed a patent claiming that modified bovine, piscine, galline, and porcine cells can grow in minimal media by ectopically expressing two or more growth factors, cytokines, or receptors (via ribosomal skipping sites) that promote cell growth, eliminating the need for exogenous growth factor supplementation^37^. Another patent was filed by Upside Foods, which used a PhiC31 integrase expression plasmid system to integrate FGF2, FGF receptors (including mutants), IGF1, and IGF1R under hEF1a promoter control into chicken fibroblasts^38^. The engineered cell lines were able to grow in growth factor-free media formulations, with expression of FGFRs surprisingly also reducing the requirement for IGF1^38^.

While genetic modification of cell lines to remove the requirement for growth factors has the potential to reduce media costs, there are several considerations for the successful implementation of this approach. Firstly, there are consumer concerns over genetic modifications. Generally, there is a lack of longitudinal studies on perceptions of GMOs for consumption, with individual studies differing in survey methodology over time. Nonetheless, some studies suggest that opposition to GMOs may be softening. The European Food Safety Authority commissioned Kantar to conduct surveys in 28 EU Member States in April 2019^39^. Bearing in mind that questions are not directly comparable, 66% of respondents surveyed in 2010 were very or fairly worried about “genetically modified organisms found in food or drink,” compared to 27% of respondents ranking it in their top five concerns towards food in the 2019 survey. Similarly, in China, there may have been a shift in public perception, with 11.9% supporting genetically modified food in 2016^40^, increasing to 55% having a positive attitude towards GM foods in 2022^41^. Interestingly, in the 2019 Eurobarometer food safety survey only 4% of respondents ranked “Genome editing” in their top five concerns^39^, perhaps reflecting a preference for the minimal use of foreign DNA made possible in gene editing.

Consequently, to increase consumer acceptance and reduce regulatory risk, groups are exploring the use of species-specific growth factors for genetic modification or performing reversible genetic modifications to remove foreign genetic material after the genes of interest have served their purpose. In a patent filed by Kent State University^42^, their technology proposes the use of *TERT* and cell-cycle genes such as *CDK4*, or even flavor-enhancing proteins like myoglobin, flanked by FRT or loxP recombination sites. After expansion to desired biomass or accumulation of myoglobin, these integrated cassettes can be removed via inducible or exogenously supplemented FLP or Cre recombinases. In another patent filed by Wildtype Inc^43^., integrated trans-differentiation-relevant genes and a dox-inducible Cre (Tet-response element promoter + Cre) are flanked by loxP sites. After differentiation, both the trans-differentiation factors and Cre can be removed with the addition of doxycycline. Meanwhile, both previously-mentioned studies^37,38^ exploring the ectopic expression of growth factors in CM cell-lines also investigated the use of species-specific growth factors, albeit under the control of foreign promoters.

Lastly, while genetic modification might suffice for eliminating GF reliance, it may not be suitable for recombinant proteins that are required in large amounts such as albumin, which is required in mg/mL concentration ranges. In a previous study, Li et al. used Piggybac transposase to integrate several different secreted protein A-fusion proteins into adherent HEK293S GnTI^-^cells^44^. While as many as 15 copies of the DNA fragment could be inserted per cell, the highest-expressing clones for each protein could only attain 8–10 mg/L concentrations. Likewise, in suspension Freestyle 293-F shake-flask cultures, they were only able to get 5-30 mg/L concentrations^44^. Therefore, this approach might not be suitable for recombinant proteins required at high concentrations, for both adherent and suspension cultures. This highlights the need for a cheaper source of recombinant proteins and growth factors.

### Production of cheaper growth factors and recombinant proteins

The recombinant production of these GFs/RPs is challenging and expensive because they require some level of post-translational modification. Consequently, despite *Escherichia coli*’s ubiquity as a recombinant protein expression system due to its ease of manipulation, speed, and cost, the commercial use of *E. coli* to directly produce soluble GFs without post-processing remains limited. After screening different GF genes, fusion partner combinations, and *E. coli* strains, Venkatesan and colleagues managed to produce a range of functionally active GFs, including FGF2, IGF1, PDGF-BB, and TGF-b1 and species-specific GFs in *E. coli*^45^. They also reported a significant reduction in the cost contribution of GFs in Essential 8 media down to 2%, from 86%, when using their own GFs. Similarly, Liu and colleagues also managed to express functional recombinant bovine FGF1 in *E. coli*, with final yields of 55 mg/L^46^.

Molecular farming in plants is gaining popularity due to its environmental friendliness, ability to scale exponentially, and improvement in productivity. The technology involves inserting DNA into host plants and using specific portions of the plants, such as leaves or seeds, as expression hosts to produce GFs and RPs. GFs/RPs have been produced in a wide variety of plant species such as rice^47^, oilseed plants^48^, tobacco^49^ and barley^50^. In interview, BioBetter claims to be able to produce insulin, transferrin, and FGF_2_ in tobacco plants and that their production cost of the protein is expected to reach $1 per gram of protein^49^. The cost of species-specific GFs produced for cultivated meat application were also generally lower as compared to PeproTech®’s growth factors from ThermoFisher Scientific (Table 3).

**Table 3:** Price of recombinant growth factors for cultivated meat industry. (Cost were obtained from the various vendors’ website on 30 Oct 2024)

Growth Factor	Vendor	Catalogue Number	Price (1 mg) (SGD$)
Porcine EGF	ORF Genetics	MK0101	35.90
Bovine/Porcine FGF-2	ORF Genetics	MK0201	136.41
Avian FGF-2	ORF Genetics	MK0202	136.41
Bovine/Porcine IGF-1 LR3	ORF Genetics	MK0301	35.90
Bovine FGF-2	Core Biogenesis	—	857.21
IGF-1 LR3	Core Biogenesis	—	268.50
EGF	Core Biogenesis	—	268.50
Bovine FGF-2	Future Fields	—	2311.10
Human FGF-2	PeproTech®	100-18B-1MG	990.00
Human EGF	PeproTech®	AF-100-15-1MG	324.00
Human IGF-1	PeproTech®	100-11-1MG	345.00

Cell-free protein expression (CFPE) systems are another way to produce cheaper GFs and RPs. These allow for quick protein expression within 24 – 48 h of addition of genetic materials as compared to days or weeks in cell-based or plant-based expression systems. The production of the GFs and RPs is also performed in a controlled environment, allowing for different conditions such as pH, and temperature to be optimized. Several reviews on the use of CFPE expression systems exist^51–53^. Common CFPE systems include E. coli extract, yeast extract, wheat germ extract, tobacco BY-2 extract, insect cell extract and mammalian cell extract. LenioBio’s BY2-based platform can yield approximately 3 g/L of growth factors and recombinant proteins^48^. Similarly, Hitachi Zosen Corporation, in collaboration with NUProtein Co. Ltd, is utilizing wheat germ cell extract for the synthesis of GFs for cultivated meat applications^54^.

## Advances to reduce cost contribution of basal media through using cheaper raw materials

To further reduce the cost of the SFM, the industry is also moving towards replacing pharmaceutical-grade media components with food-grade alternatives. A cost analysis conducted by Liz Specht highlighted that replacing basal medium components with bulk, food-grade, equivalents could reduce basal media cost by 77%^9^. Food-grade components are on average 82% cheaper compared to their reagent-grade alternatives at 1 Kg scale (Table 4). Pharmaceutical-grade materials are costlier due to their high purity and certification by rigorous quality standards, including additional purification and endotoxin testing not typically performed for food-grade materials^55^. Consequently, food-grade materials may have greater batch variability and contaminants, thus necessitating separate testing before use. For instance, Stellavato et al. found that of the ten chondroitin sulfate and glucosamine food supplements they tested, none of them contained their declared concentrations of both substances; all were contaminated with the structurally-similar keratan sulfate; and most were cytotoxic to their cell models^56^.

**Table 4:** Price comparison between reagent-grade media component and food-grade media component. (Cost were obtained from Sigma-Aldrich website on 30 Oct 2024)

	Catalogue Number	Reagent-grade price (1 Kg) (SGD$)	Catalogue Number	Food-grade price (1 Kg) (SGD$)	Food-grade/ Reagent-grade (%)
L-Arginine	A8094	489	W381920	149	30.5%
L-Cysteine	C7352	1920	W326305	444	23.1%
L-Isoleucine	I7403	2960	W527602	428	14.5%
L-Leucine	L8912	1470	W329703	217	14.8%
L-Phenylalanine	P5482	1980	W358512	335	16.9%
L-Proline	P5607	1840	W331902	230	12.5%
L-Tyrosine	T8566	1760	W373605	286	16.3%
Thiamine hydrochloride	T1270	2790	W332208	256	9.18%
L-Ascorbic acid	A4544	570	W210901	134	23.5%
				Average	17.9%

Despite this, food-grade alternatives like plant hydrolysates are more cost-effective, still safe for human consumption, and nutrient-rich, containing bioactive compounds beneficial for animal cell culture. They are commonly used to reduce the amount of serum required^57^, and when used appropriately, can even replace traditional basal media by serving as a source of carbon and nitrogen^5^. The bioactive peptides found in hydrolysates can promote cell growth and offer additional benefits like anti-apoptotic, antioxidant, immunomodulatory properties, and potentially substituting growth factors^5,58,59^. Hydrolysates also lower storage and sterilization costs due to their heat stability^5,57^ and can enhance the flavor and nutritional profile of cultivated meat^5,60^. Humbird also estimated that the use of plant hydrolysate will be able to reduce the cost of amino acids down to $2 per Kg of mixed amino acids^7^.

Some cultivated meat companies have reported success in replacing pharmaceutical-grade media components with food-grade alternatives. For example, Mosa Meat, in partnership with Nutreco, replaced 99.2% of the basal cell feed by weight with food-grade components, while achieving cell growth comparable to pharmaceutical-grade media^61^. Similarly, Nutreco and Blue Nalu demonstrated that suspension bluefin tuna muscle-derived cells grew equally well in both food-grade and pharmaceutical-grade cell culture media^62^. A study by IntegriCulture Inc. demonstrated that mouse skeletal muscle-derived cells (C2C12) and bovine skeletal muscle-derived primary cells (BSMCs) can grow in food-grade DMEM^63^. Additionally, IntegriCulture Inc., together with JT Group, reduced 31 media components to 16 by replacing some amino acid components with yeast extract^64^. This food-grade I-MEM2.0 could support cell growth from various cell types and species, including bovine skeletal muscle-derived primary cells, duck liver-derived cells, and five different chicken primary cells^64^.

There are already comprehensive reviews on the use of hydrolysates in animal cell culture, particularly those derived from non-animal sources such as soy, wheat, rice and yeast^57–59,65^. In the following section we will focus on advances in the use of hydrolysates from other sources, such as microalgae extracts and agricultural side streams for cultivated meat application.

### Microalgae Extracts

Research on the use of microalgae extracts as cell culture supplements is gaining interest due to their environmental friendliness and high nutritional content, including proteins, fatty acids, trace elements, and vitamin B^66,67^. Extracts from *Chlorella vulgaris* have been shown to support the proliferation of embryonic bovine tracheal fibroblasts^68^ and the proliferation and differentiation of primary bovine myoblasts^69^. Other microalgae are also being investigated. For instance, *Chlorococcum littorale’s* extracts have been found to promote cell growth in C2C12, 3T3, and Chinese hamster ovary (CHO) cell lines in serum-free environments^70^. Additionally, Cyanobacteria *Anabaena sp. PCC 7120*’s extract was used as a media supplement to cultivate mouse and quail muscle cells^71^. Next, *Auxenochlorella pyrenoidosa* protein extract – together with l-ascorbic acid, insulin, transferrin, selenium, and ethanolamine – was successfully used as FBS replacement for *Carassius auratus* (goldfish) muscle cell proliferation^72^. Despite their potential, the nutritional profiles of microalgae have not been thoroughly studied, with different algae species varying in their nutritional content. Future research could investigate other microalgae with Generally Recognized as Safe (GRAS) status, to evaluate their suitability as media supplements for cultivated meat applications^73^.

### Agricultural side streams

To develop cost-effective media component replacements, researchers are also exploring the use of agricultural side streams for cellular agriculture. Efficient side stream valorization allows for the better utilization of agricultural byproducts, resulting in less waste and promoting a circular bioeconomy with lower overall monetary and environmental costs compared to FBS usage^74,75^. Various groups have attempted to use soybean meal as partial FBS replacement^76–78^. Kim and colleagues demonstrated that they could partially replace FBS with fermented soybean meal and edible insect hydrolysates for the cultivation of pig muscle stem cells^76^. Similarly, Teng and colleagues showed that okara extracts could partially replace FBS during the cultivation of C2C12 and immortalized porcine myoblasts^78^, although dose-dependent toxicity was observed. Additionally, rapeseed protein isolates from agricultural waste were found to be capable of replacing albumin used in Beefy-9 for the cultivation of bovine satellite cell cultures^79^. Hydrolysates from various animal-based food processing byproducts and yeast extract were also shown to support bovine skeletal muscle cells in terms of cell growth and function, with hydrolysates consisting of peptides 2 – 15 amino acids-long improving cell growth and increasing cellular metabolism^80^. Ultimately, while the utilization of side streams could result in less waste and lower cost, proper side stream management must be implemented to prevent unwanted contaminants.

### Considerations for using hydrolysates as food-grade media alternative

Several issues must however be addressed before hydrolysates can be effectively used in cultivated meat production^5^. Variations in hydrolysate quality can arise from factors such as differences in raw materials, species, storage conditions, and processing methods^5,57,58^. Optimization of hydrolysates’ processing methods, well-defined and rigorous quality control testing methods, specifications for raw materials, qualitative and quantitative analysis of hydrolysate end-product are thus needed, to minimize batch-to-batch variation in cultivated meat production^58,65,81^. More studies will also need to be conducted to assess the effect of inter-batch variation of hydrolysates on cell mass, metabolism, and differentiation.

Secondly, protein extracts may contain impurities such as heavy metals, pesticides, cytotoxic and allergenic components^60,75^. Group 2B compounds, possibly carcinogenic compounds such as free and esterified forms of 3-monochloro-1,2-propanediol and 1,3-dichloro-1,2-propanol, are reported to be found in acid-hydrolyzed vegetable proteins^60^. Furthermore, the use of nut-based hydrolysates might have immunological cross-reactivity in peanut-allergic consumers^60^. To address these safety concerns, hydrolysates can be produced from GRAS organisms or traditional crops/foods with a history of human consumption. Future studies should also investigate the accumulation of impurities in cultivated meat cells and their impact on human consumption.

Finally, while hydrolysates can support cell growth, they still lack some nutrients compared to FBS. Identifying and supplementing these missing nutrients is essential to achieving better growth than FBS-containing cultures and to fully replace FBS and current basal media^71^. Effects of hydrolysates as serum replacement are also cell-line dependent^57^. More research is needed for industry-wide adoption of hydrolysates as pharmaceutical-grade media replacements.

## Increasing the longevity of the serum-free media for cost reduction

Another way to reduce the cost of media, and labour costs associated with media-change operations, will be to increase the usability of the media for a longer period. A comprehensive review was published by Yang et al. on preventing waste buildup through genetic engineering techniques, cell culture strategies, affinity removal methods, biocatalytic methods, and electrochemical methods^82^. In this section, we will discuss preventing waste buildup through the optimization of media components, improving GF stability and other media recycling techniques.

### Optimization of media components

Ammonia, a potent inhibitor of cell growth, is produced during glutamine metabolism. To reduce ammonia production during the proliferation and adipogenesis of fibro-adipogenic progenitors (FAPs), Hubalek et al. developed novel serum-free media for both proliferation and differentiation by replacing GlutaMAX with non-ammoniagenic compounds like α-ketoglutarate (aKG), glutamate, and pyruvate, which are involved in the TCA cycle or glutaminolysis^83^. They showed that pyruvate and aKG led to comparable cell growth rates with no ammonia produced during short-term proliferation^83^. FAPs were also able to maintain their differentiation capacity when Glutamax was replaced with aKG, pyruvate and glutamate, with a 2.1-fold increase in adipogenic capacity in cells grown and differentiated in non-ammoniagenic media^83^.

The substitution of glutamine by other components to reduce ammonia buildup was also successfully demonstrated in other commercial cell lines^84,85^. Replacing glutamine with TCA intermediates like aKG, citric acid, and succinic acid in recombinant CHO cell cultures resulted in similar growth rates after a lag phase and significantly reduced ammonia production^84^. Cell growth of various mammalian cell types Madin-Darby canine kidney cells, Baby Hamster kidney fibroblasts and CHO cells was also supported for at least 19 passages in media containing pyruvate as a substitution for glutamine, with no ammonia accumulation^85^.

Other than ammonia production, media optimization could also be performed to reduce production of lactate^86,87^, another potent inhibitor of cell growth that arises from glycolysis and amino acid catabolism. To that end, our lab demonstrated that using maltose as an alternative energy source to glucose for CHO cells and HEK293 cells resulted in lower levels of lactate, which could potentially improve cell and protein productivity^88^. However, other disaccharides failed to sustain cell growth^88^. Similarly, Buchsteiner et al. were able to reduce lactate production, without adverse effects, in CHO cells by supplementing the media with dichloroacetate, an inhibitor of pyruvate dehydrogenase kinase^89^.

Another way to prolong the media lifespan is to stabilize the growth factors used. For example, the common GF supplement, FGF2, was found to be stabilized and prevented from heat-induced aggregation by addition of its known cofactor, heparin^90^. Alternatively, thermostable FGF2 mutants, such as FGF2-G3 with nine point mutations to increase its half-life from 10 h to 20 days^91^, have successfully been used in the culture of induced pluripotent stem cells to lower media change frequencies^14^. Similarly, thermostable mutants of FGF1^92^ and FGF7^93^ have been reported, although work on other GFs such as IGF1 and EGF is lacking.

### Media Recycling

Although the concept of media recycling to reduce media costs first started in 1977^94,95^, Believer Meats is the only cultivated meat company to implement spent media recycling technology to date^96^. In 2017, Yaakov Nahmias, Believer Meats’ founder, submitted a patent application for a closed-loop perfusion bioreactor system^96^. This system enables the recycling of media by guiding it through a dialyzer that filters out harmful metabolites, retaining large proteins such as albumin and replenishes nutrients before circulating back to the tissue growth chambers where cells are cultivated

Media recycling has also been applied in areas beyond cultivated meat production. Masahiro Kino-oka’s team proposed a dialysis setup to remove lactate and to recycle autocrine factors and GFs for the culturing of human induced pluripotent stem cells in suspension^97^. With this setup, cells could be maintained with lower concentrations of supplemented TGF-β1, insulin, transferrin, L-ascorbic acid, and selenium. In another study by Akiko Ogawa’s team, media recycling was achieved by passing spent media through an affinity protein G column, concentrating it by ultrafiltration, then diluting it with fresh basal medium^98^. In doing so, they saved 67% of the complete media for growing IgG1 antibodies producing hybridoma cells. In a similar study by Lin’s team, the permeate from perfusion CHO cultures used for monoclonal antibody production was successfully recycled by passing sequentially through a Protein A column and cation exchange depth filter to remove process impurities, then mixed with concentrated media^99^. They, however, cautioned that since their recycled stream required mixing with media concentrate and subsequent osmolality balancing to retain media depth and cell proliferation, only water was effectively being saved, with little material cost benefits^99^.

Microalgae are also being explored for renewing spent mammalian culture media by removing waste like ammonia and lactate and replenishing depleted nutrients via microalgae extract supplementation. For instance, *Chlorococcum littorale* removes over 90% of ammonia while restoring glucose and amino acids^100^. To enhance this process, Yuji and his team used recombinant *Synechococcus sp*. PCC 7002 that can utilize both ammonia and lactate^101^. However, nutrient levels in microalgae extracts can vary, requiring adjustments to the microalgae extract added and growth factors need supplementation after several media renewal cycles^100,101^.

## Conclusion

In conclusion, cultivated meat production holds immense promise for addressing global food security and sustainability challenges. However, the current production process remains expensive, primarily due to the high cost of SFM. Achieving price parity with conventional meat requires the development of scalable, low-cost culture media. This review has explored the current technologies and opportunities for creating cost-effective SFM tailored to cultivated meat production. Key strategies include reducing the costs associated with growth factors and recombinant proteins and lowering the expense of basal media by utilizing more affordable raw materials. Additionally, innovative approaches such as media recycling and waste reduction were discussed as alternative cost-saving measures. While this review focused on economic aspects, it is also important to recognize these strategies could also reduce the environmental impact of cultivated meat productions. Other critical factors in media development, such as the impact of culture media on the organoleptic and nutritional qualities of cultivated meat products, were beyond the scope of this review but remain vital areas for future research.

## Supplementary information

Supplementary Information

## Data Availability

No datasets were generated or analysed during the current study.
