# Supplementary material for: Exploring cost reduction strategies for serum free media development
Source: NPJ Sci Food. 2024 Dec 21;8:107. doi: 10.1038/s41538-024-00352-0 (PMC11663224; doi:10.1038/s41538-024-00352-0)
Supplement: Supplementary file 1 — Supplementary Information [file 41538_2024_352_MOESM1_ESM.pdf]

Table S1. Cost of media component and price analysis of growth media for primary bovine satellite cells developed by Kolkmann et al[8]. (Cost were obtained from the respective supplier on 24 May 2024)

Lab scale								
**Component**	**Unit/L**	**Amount/L**	**Supplier**	**Cat. No**	**Units/order**	**Cost/order**	**Cost/L**	**Percentage of total cost/%**
**Basal Media**								
DMEM/F-12	ml	1000	ThermoFisher	21331020	500	107	214	3.77
**Proteins/Peptides**								
VEGF	ug	10	R&D systems	BT-VEGF-AFL-050	50	1350	270	4.76
Fibronectin	mg	10	Sigma-Aldrich	10838039001	5	843	1686	29.69
Human IL-6	ug	20	PeproTech	200-06-20UG	20	317	317	5.58
IGF-1	ug	100	R&D systems	291-G1-200	200	590	295	5.20
HGF	ug	5	R&D systems	294-HGN-005/CF	5	620	620	10.92
PDGF-BB	ug	10	R&D systems	220-BB-010	10	710	710	12.50
FGF-2	ug	10	R&D systems	Qk002-0100	100	480	48	0.85
Human serum albumin	g	5	Sigma-Aldrich	A9511-5G	5	1040	1040	18.32
**Steroid/Hormone/Drug**								
Hydrocortisone	ug	36	Sigma-Aldrich	H0888-1G	10000000	109	0.000392	0.00
**Nutrient/Buffer/Salts**								
α-linolenic acid	mg	1	Sigma-Aldrich	L-039-1ML	1	269	269	4.74
GlutaMAX	ml	10	ThermoFisher	35050061	100	129	12.9	0.23
L-ascorbic acid 2-phosphate	mg	50	Sigma-Aldrich	49752-10G	10000	182	0.91	0.02
**Mixtures**								
ITS-X	ml	10	ThermoFisher	51500056	10	195	195	3.43
						**SGD**	**5677.81**	**100.00**

Table S2. Cost of media component and price analysis of growth media for primary bovine satellite cells developed by Stout et al[10]. (Cost were obtained from the respective supplier on 24 May 2024)

Lab scale								
**Component**	**Unit/L**	**Amount/L**	**Supplier**	**Cat. No**	**Units/order**	**Cost/order**	**Cost/L**	**Percentage of total cost/%**
**Basal Media**								
DMEM/F12 + HEPES + Sodium bicarbonate	mL	1000	ThermoFisher	11330032	500	106	212	27.0
**Proteins/Peptides**								
rAlbumin	mg	800	Sigma	A9731-1G	1000	255	204	26.0
Insulin	mg	20	Sigma	91077C-250MG	250	513	41.04	5.2
Transferrin	mg	20	Sigma	T8158-100MG	100	288	57.6	7.3
FGF-2	ug	40	Peprotech	100-18B-50UG	50	323	258.4	32.9
NRG1	ng	100	Peprotech	100-03-10UG	10000	131	1.31	0.2
TGFb3	ng	100	Peprotech	100-36E-2UG	2000	131	6.55	0.8
**Nutrient/Buffer/Salts**								
L-ascorbic acid 2-phosphate	mg	200	Sigma	49752-10G	10000	182	3.64	0.5
Sodium selenite	ug	20	Sigma	S5261-10G	10000000	81.2	0	0.0
						**SGD**	**784.54**	**100.0**

Table S3. Cost of media component and price analysis of growth media for primary bovine satellite cells developed by Skrivergaard et al[11]. (Cost were obtained from the respective supplier on 24 May 2024)

Lab scale								
**Component**	**Unit/L**	**Amount/L**	**Supplier**	**Cat. No**	**Units/order**	**Cost/order**	**Cost/L**	**Percentage of total cost/%**
**Basal Media**								
DMEM/F12 + HEPES + Sodium bicarbonate	mL	1000	ThermoFisher	11330032	500	106	212	36.18
**Proteins/Peptides**								
FGF-2	ug	2	Future Fields, Bovine FGF2, EntoEngine, purified version	-	50	150	6	1.02
Fetuin	mg	600	Sigma-Aldrich	F2379-1G	1000	391	234.6	40.03
BSA	mg	75	Sigma-Aldrich	A8412-100ML	7500	141	1.41	0.24
**Mixtures**								
ITS (1X)	ml	10	ThermoFisher	41400045	10	132	132	22.53
						**SGD**	**586.01**	**100**

Table S4. Cost of media component and price analysis of growth media for primary chicken fibroblasts cells developed by Pasitka et al[25]. (Cost were obtained from the respective supplier on 30 May 2024)

Lab scale								
**Component**	**Unit/L**	**Amount/L**	**Supplier**	**Cat. No**	**Units/order**	**Cost/order**	**Cost/L**	**Percentage of total cost/%**
**Basal Media**								
DMEM/F-12	mL	1000	Gibco	21331-020	500	107	214	37.98
**Proteins/Peptides**								
Insulin	mg	3	Sigma-Aldrich	I0516-5ML	50	261	15.66	2.78
FGF	ug	10	Peprotech	100-18B-100UG	100	356	35.6	6.32
**Steroid/Hormone/Drug**								
Hydrocortisone	mg	2	Sigma-Aldrich	H0135-1MG	1	140	280	49.69
**Nutrient/Buffer/Salts**								
Sodium selenite	ug	7	Sigma-Aldrich	S5261-10G	10000000	32.1	0	0.00
L-alanine-L-glutamine	g	0.43	Sigma-Aldrich	A8185-5G	5	178	15.46	2.74
**Mixtures**								
Canola lipid mixture	mg	10	Sigma-Aldrich	PHR2971-1G	1000	277	2.77	0.49
						**SGD**	**563.49**	**100.00**

Table S5. Cost of media component and price analysis of differentiation media for Primary Bovine Satellite Cells developed by Messmer et al[16]. (Cost were obtained from the respective supplier on 12 June 2024)

**Lab scale**								
**Component**	**Unit/L**	**Amount/L**	**Supplier**	**Cat. No**	**Units/order**	**Cost/order**	**Cost/L**	**Percentage of total cost/%**
**Basal Media**								
DMEM/F-12	mL	1000	ThermoFisher	21331020	500	107	214	47.11
**Proteins/Peptides**								
Insulin	mg	10.44	Sigma-Aldrich	91077C-100MG	100	270	28.19	6.21
Transferrin	mg	10.80	Sigma-Aldrich	T3705-1G	1000	749	8.09	1.78
Human serum albumin	g	0.50	Sigma-Aldrich	A9511-1G	1	311	155.50	34.23
EGF-1	µg	10.00	Peprotech	AF-100-15-100UG	100	135	13.50	2.97
**Nutrient/Buffer/Salts**								
L-Ascorbic acid 2-phosphate	mg	11.58	Sigma-Aldrich	A8960-5G	5000	158	0.37	0.08
Sodium selenite	µg	13.84	Sigma-Aldrich	S5261-10G	10000000	81.2	0.00	0.00
Sodium bicarbonate	g	0.69	Sigma-Aldrich	S2127-1KG	1000	212	0.15	0.03
Lysophosphatidic acid	mg	0.44	Sigma-Aldrich	L7260-5MG	5	394	34.40	7.57
**Mixtures**								
MEM Amino acid solution	mL	5	Sigma-Aldrich	M5550-100ML	5000	69.9	0.07	0.02
						**SGD**	**454.26**	**100.00**

Table S6. Cost of media component and price analysis of differentiation media for Bovine, porcine, and murine adipogenic precursor cells developed by Mitić et al[21] (Cost were obtained from the respective supplier on 12 June 2024)

Lab scale								
**Component**	**Unit/L**	**Amount/L**	**Supplier**	**Cat. No**	**Units/order**	**Cost/order**	**Cost/L**	**Percentage of total cost/%**
**Basal Media**								
DMEM/F-12	mL	1000	ThermoFisher	21331020	500	107	214.00	43.20
**Proteins/Peptides**								
FGF-2	µg	2	Peprotech	100-18B-100UG	100	356	7.12	1.44
EGF1	µg	2	Peprotech	AF-100-15-100UG	100	135	2.70	0.55
Insulin	mg	10	Sigma-Aldrich	I0516-5ML	50	261	52.20	10.54
BMP4	µg	10	Peprotech	120-05ET-100UG	100	1609	160.90	32.48
**Steroid/Hormone/Drug**								
Progesterone	µg	5.60	Sigma-Aldrich	P8783-1G	1000000	60.9	0.00	0.00
Hydrocortisone	µg	9.06	Sigma-Aldrich	H0135-1MG	1000	140	1.27	0.26
Rosiglitazone	mg	1.79	Sigma-Aldrich	R2408-10MG	10	224	40.03	8.08
**Nutrient/Buffer/Salts**								
L-Ascorbic acid 2-phosphate	mg	65.73	Sigma-Aldrich	A8960-5G	5000	158	2.08	0.42
Glucose	g	3.06	Sigma-Aldrich	G7021-100G	100	89.8	2.75	0.56
Putrescine	mg	5.02	Sigma-Aldrich	51799-100MG	100	86.7	4.36	0.88
Calcium chloride	g	0.15	Sigma-Aldrich	C3881-500G	500	162	0.05	0.01
HEPES	g	1.17	Sigma-Aldrich	H4034-25G	25	127	5.93	1.20
**Mixtures**								
Lipid concentrate	mL	1	Gibco	11905-031	100	193	1.93	0.39
						**SGD**	**495.31**	**100.00**
